# Culture expansion of CAR T cells results in aberrant DNA methylation that is associated with adverse clinical outcome

**DOI:** 10.1038/s41375-023-01966-1

**Published:** 2023-07-14

**Authors:** Lucia Salz, Alexander Seitz, Daniel Schäfer, Julia Franzen, Tatjana Holzer, Carlos A. Garcia-Prieto, Iris Bürger, Olaf Hardt, Manel Esteller, Wolfgang Wagner

**Affiliations:** 1 Institute for Stem Cell Biology, RWTH Aachen University Medical School, Aachen, Germany; 2 Helmholtz-Institute for Biomedical Engineering, RWTH Aachen University Medical School, Aachen, Germany; 3 Miltenyi Biotec B.V. & Co. KG, Bergisch, Gladbach Germany; 4 Josep Carreras Leukemia Research Institute (IJC), Badalona, Barcelona, Catalonia Spain; 5 Life Sciences Department, Barcelona Supercomputing Center (BSC), Barcelona, Spain; 6 Centro de Investigacion Biomedica en Red Cancer (CIBERONC), Madrid, Spain; 7 Institucio Catalana de Recerca i Estudis Avançats (ICREA), Barcelona, Catalonia Spain; 8 Physiological Sciences Department, School of Medicine and Health Sciences, University of Barcelona (UB), Barcelona, Catalonia Spain

**Keywords:** Translational research, Cell signalling, Immunotherapy, Genomics

## Abstract

Chimeric antigen receptor (CAR) T cells provide new perspectives for treatment of hematological malignancies. Manufacturing of these cellular products includes culture expansion procedures, which may affect cellular integrity and therapeutic outcome. In this study, we investigated culture-associated epigenetic changes in CAR T cells and found continuous gain of DNAm, particularly within genes that are relevant for T cell function. Hypermethylation in many genes, such as *TCF7*, *RUNX1*, and *TOX*, was reflected by transcriptional downregulation. 332 CG dinucleotides (CpGs) showed an almost linear gain in methylation with cell culture time, albeit neighboring CpGs were not coherently regulated on the same DNA strands. An epigenetic signature based on 14 of these culture-associated CpGs predicted cell culture time across various culture conditions. Notably, even in CAR T cell products of similar culture time higher DNAm levels at these CpGs were associated with significantly reduced long-term survival post transfusion. Our data demonstrate that cell culture expansion of CAR T cells evokes DNA hypermethylation at specific sites in the genome and the signature may also reflect loss of potential in CAR T cell products. Hence, reduced cultivation periods are beneficial to avoid dysfunctional methylation programs that seem to be associated with worse therapeutic outcome.

## Introduction

Cellular immunotherapy using autologous, genetically modified T cells has delivered remarkable initial response rates in various hematological malignancies, including acute lymphoblastic leukemia (ALL) or lymphoma [1]. Upon treatment with these chimeric antigen receptor (CAR) T cells, approximately 30–40% of patients show long-term remission [2]. There is a growing perception that not only CAR constructs, but also in vitro manufacturing processes may determine therapeutic efficacy. CAR T cell numbers in a formulated drug product usually range between 10^6^ and 10^7^ cells per kilogram of body weight, which demands culture expansion [3]. However, little is known about how duration of culture expansion affects molecular and functional features of CAR T cells. A better understanding of molecular signatures, reflecting therapeutic activity of CAR T cells, may help to further optimize the manufacturing process [4].

DNA methylation (DNAm) at specific CG dinucleotides (CpG sites) in the genome is one of the most relevant epigenetic processes. It is mediated by epigenetic writers–such as the de novo methyltransferases DNMT3A and DNMT3B, or TET methylcytosine dioxygenases [5]. Several fundamental details about the precise regulation of DNAm patterns during development and their functional implications remain to be elucidated [6]. Either way, DNAm is highly consistently modified between cell types, during aging, and upon malignant transformation, making it a well suited biomarker for diagnostic applications [7]. A recent study has analyzed the epigenetic landscape of pre-infusion CAR T cells directed against CD19. The authors identified DNAm changes caused by the transduction of cells with the CAR vector. Notably, their epigenetic signature, called EPICART, was associated with complete response (CR), event-free survival (EFS), and overall survival (OS) post infusion [8]. So far, it is largely unclear how DNAm of CAR T cells changes with time in cell culture, independent from the transduction with the viral CAR vectors.

In vitro expansion of stem and progenitor cells seems to be generally associated with a continuous functional decline and ultimately proliferation arrest– a state, referred to as replicative senescence. We and others have previously shown that culture expansion of mesenchymal stromal cells (MSCs), fibroblasts, and hematopoietic stem and progenitor cells (HSPCs) is associated with highly reproducible DNAm changes, which may be caused by a stochastic process, referred to as “epigenetic drift” [9]. Long-term culture-associated DNAm changes could be used to predict time in culture or cumulative population doublings [10]. Importantly, the culture-associated DNAm changes are related, but not identical, to the so called “epigenetic clocks” that reflect aging of the organism [9, 11]. Furthermore, there are cell-type specific differences in DNAm changes during in vitro expansion.

In this study, we therefore investigated DNAm changes during culture expansion of T cells and CAR T cells. We demonstrate that several CpGs become continuously hypermethylated, particularly within genes that are related to T cell function and that have previously been associated with T cell exhaustion in vivo. Due to the stochastic pattern of DNAm at neighboring CpGs, the process does not seem to be directly regulated, but rather resembles a stochastic drift. These DNAm changes were not only indicative for the duration of cultivation before infusion, but also capture CAR T cell product intrinsic variation associated with long-term survival post transfusion.

## Materials and methods

### Ethics approval and consent to participate

All blood donors and patients provided written informed consent according to the Declaration of Helsinki and the International Conference on Harmonization Guidelines for Good Clinical Practice. Isolation of T cells from peripheral blood from healthy donors was performed according to the guidelines approved by the local ethics committees of RWTH Aachen University (EK 041/15). The clinical trials NCT03853616 and NCT03870945 were approved by the Paul-Ehrlich-Institut (Germany). All blood samples were handled following the required ethical and safety procedures.

### Blood samples

T cells were isolated from peripheral blood from healthy donors. For generation of CAR T cells, we used either buffy coats or leukapheresis products from healthy donors that were purchased from the German Red Cross Dortmund or DRK Ulm and MHH Hannover, respectively. Furthermore, we analyzed CAR T cells that were obtained from fresh non-mobilized leukapheresis products from patients with acute lymphoblastic leukemia (ALL) or diffuse large B cell lymphoma (DLBCL) that were enrolled in clinical trials of MB-CART19.1 or MB-CART2019.1 (NCT03853616 and NCT03870945).

### Cell culture

In this study, we compared different cell culture regimen: For expansion of T cells with CD2/CD3/CD28-loaded particles, we isolated peripheral blood mononuclear cells (PBMCs), selected CD3^+^ cells with the MicroBead Kit (Miltenyi Biotec), and confirmed successful enrichment by flow cytometry. T cells (10^6^ cells/mL) were resuspended in RPMI (Gibco Thermo Fisher), 10% FCS (Gibco Thermo Fisher), 20 U IL-2 per mL (Miltenyi Biotec), with the T Cell Activation/Expansion Kit, human (containing CD2/CD3/CD28-loaded MACSiBead Particles; Miltenyi Biotec) according to manufacturer’s instructions. Restimulation was performed at day 7 and day 17. At every passage, cells were counted with a Neubauer chamber to calculate cumulative population doublings.

For expansion of T cells/CAR T cells with CD3/CD28-conjugated polymeric nanomatrix we selected T cells with the human Pan T Cell Isolation Kit (Miltenyi Biotec) and validated enrichment by flow cytometry. The cells were resuspended in TexMACS™ Medium (Miltenyi Biotec) supplemented with recombinant human IL-7 (12.5 ng/mL) and IL-15 (12.5 ng/mL, both Miltenyi Biotec), and activated with polymeric nanomatrix conjugated to humanized CD3 and CD28 (T Cell TransAct^TM^; Miltenyi Biotec).

For automated expansion of T cells all steps of cell enrichment, lentiviral CAR transduction of T cells as well as cultivation and media exchanges were performed on the CliniMACS Prodigy^®^ (Miltenyi Biotec). In detail, CD4^+^ and CD8^+^ T cells from leukapheresis products of healthy donors and patients were enriched by magnetic positive selection, directly followed by T cell activation using T Cell TransAct^TM^ (Miltenyi Biotec). T cells were transduced after 24 h using a lentiviral vector expressing either a CAR against human CD19 or a tandem CAR against human CD20 and CD19 (Lentigen). T cells were expanded in TexMACS GMP medium supplemented with IL-7, IL-15 and 3% human AB serum at 5% CO_2_ and 37 °C. After 5 days, the medium was replaced by TexMACS GMP medium supplemented with IL-7 and IL-15. Harvest and formulation of cells was performed after 12 days using Composol or CliniMACS Formulation Solution, supplemented with 2.5% human AB serum (HSA).

### Generation of CAR constructs and lentiviral vectors

For manual cultured small scale experiments we used the anti-LLE biotin adapter CAR (AdCAR). In brief, an anti-LLE scFv was cloned on a third generation backbone. The scFv was preceded by an IL-22 leader sequence and followed by a CD8α hinge and transmembrane domain. Intracellular domains consisted of CD28 followed by 4–1BB as co-stimulatory domains and a CD3ζ main-stimulatory domain. Lentiviral particles were resuspended in TexMACS and stored at −70 °C until further use. T cells were transduced 24 h after activation and culture was continued following the recommended T Cell TransAct^TM^ protocol.

### Flow cytometry

For immunophenotypic analysis of initial cell preparations and during culture expansion, the cells were stained (concentration 1:50; 10 min, at 4 °C) against CD3 (clone REA613), CD4 (clone REA623), CD8 (REA734), CD45RO (clone REA611), CD62L (clone REA615), CD95 (clone REA738), and CAR (Anti-Biotin-PE clone REA746; all Miltenyi Biotec). After incubation, a wash step was performed with AutoMACS Running Buffer to the suspension, and the fluorescence was measured on a MACSQuant Analyzer 10 with MACSQuantify™ Software v2.13.0.

### DNA methylation profiling

Genomic DNA (gDNA) was isolated with the NucleoSpin Tissue kit (Macherey-Nagel) or DNeasy Blood & Tissue Kit (Qiagen), bisulfite converted, and hybridized to the Infinium MethylationEPIC BeadChips (Illumina, San Diego, California, USA) at the Life and Brain GmbH (Bonn, Germany). Raw data was preprocessed with the Bioconductor Illumina Minfi package for R and normalized with ssNoob [12]. For further analysis, we excluded CpGs with common single nuclear polymorphisms (SNPs; MAF > 1%), CpGs of X and Y chromosomes, and probes that were identified as cross-reactive. Since the CD4^+^/CD8^+^ ratio changes during culture expansion, we also filtered for CpGs that were significantly differentially methylated between purified CD4^+^ and CD8^+^ cells (GSE110554): Thereby 46,856 CpGs with a mean DNAm difference of >10% or an adjusted *p* < 0.05 (limma t-test) between CD4^+^ and CD8^+^ cells were excluded.

Principal component analysis was performed in R and plotted with the ggplot2 version 3.3.6 package. Volcano plots were generated with the EnhancedVolcano R package version 1.12.0. Gene ontology analysis was performed on hypo- and hypermethylated CpG sites (DNAm change in T cells at day 0 *versus* day 22 in culture of more than 20% and adjusted *p* < 0.05) in R with the missMethyl package. Heatmaps were generated with the ComplexHeatmap R package. Information of the Illumina BeadChip annotation file was used to define promoter categories (TSS1500, TSS200, and 5’UTR) and gene body regions (Body, 1stExon, and 3’UTR). To estimate if culture-associated DNAm changes in T cells are similar in other cell types, we used available datasets of CD34^+^ cells from cord blood [13], and HUVECs [14], and mesenchymal stem cells [15], even though these cells were cultured for different times and under different culture conditions. Since, all of these datasets were generated on 450 K Illumina BeadChips we focused on CpGs that are common in the 450 K and EPIC platforms, while batch variation between studies and platforms cannot be excluded.

### Epigenetic predictor for time in culture

Culture-associated CpGs were initially selected based on Pearson correlation of DNAm levels (β values) and days in culture at a threshold of *r* > 0.9 or *r* < −0.9 (336 hyper- and 3 hypomethylated CpGs, respectively) in a training set of 15 healthy donors. To exclude age-associated CpGs, which may rather be affected by donor age than by culture expansion, we used a dataset of peripheral white blood cells (GSE115278; *n* = 474; mean age: 47.2 ± 14.1 years). Here, 7 out of 339 CpGs correlated with chronological age (*r* > 0.3 or *r* < −0.3) and were removed. The predictor for time in culture was then designed as linear regression model fitting the elastic-net regularization path in the training dataset using the glmnet R package [16]. An elastic-net regression model was applied to the 332 candidate CpGs with the mixing parameter set to α = 0.5. Best-fitted CpGs and their coefficients were chosen by 10-fold cross-validation (Supplemental Tables S1).

### Epigenetic predictor for loss of T cell potential

The association of culture-time associated DNAm with therapeutic outcome was tested on DNAm profiles of CAR T cell products from three clinical trials (NCT02772198, NCT03144583, and NCT03373071) including patients with relapsed and/or refractory (r/r) Non-Hodgkin lymphoma (NHL) or r/r B cell acute lymphoblastic leukemia (ALL) [8]. Multivariate Cox proportional hazards regression models were generated with the survival R package version 3.4–0. Initially we considered the available covariates ‘origin of clinical trial’, ‘disease type’ (ALL/NHL), ‘age’, and ‘gender’ to train a model for the 332 culture-associated CpGs. However, since the covariates ‘age’ and ‘gender’ did not have a significant impact, we performed stepwise reduction of these. Only ‘origin of clinical trial’ and ‘disease type’ were included as covariates in the final Cox models. The ß-values have been applied as continuous variables to the Cox model and the hazard ratio (HR) is to be interpreted per 10% DNAm difference.

Next, CpG sites with an overall survival association of *p* < 0.01 (14 CpGs) were selected as candidates in an subset of GSE179414 for target identification. With these 14 CpGs, a linear regression model to predict time in culture, fitting the ridge regularization path, was designed in an independent, preclinical training dataset. Then, the Cox model, adjusted for ‘origin of clinical trial’ and ‘disease type’, was applied to calculate HRs for the culture time predictions of the ‘Ridge 14 CpG Predictor’. The HR is to be interpreted per day difference of predicted time in culture. Survival curves were plotted with the survminer R package version 0.4.9. The level of significance is indicated with *p*-values calculated with Wald test based on Cox model.

### Gene expression analysis

Total RNA was isolated from freshly isolated T cells (day 0) and upon three weeks of culture (day 22) in IL-2 supplemented medium with the NucleoSpin RNA Plus kit (Macherey-Nagel). Library preparation with the QuantSeq 3’ mRNA-Seq Library Prep Kit FWD (Lexogen, Vienna, Austria) and sequencing on the HiSeq 2500v4 platform (Illumina; 10 M 50 bp single-end reads) was performed at Life and Brain GmbH. RNA sequencing (RNA-seq) reads were aligned with the R package QuasR and Rhisat2 as aligner to the human genome (hg19) with default parameters [17, 18]. Reads that aligned with less than 10 reads per gene were excluded from further analysis and counts per million (CPM) were calculated and TMMwsp normalized with standard parameters using the R package edgeR [19]. Count data was voom-transformed by applying the R package limma [20]. For differential expression analysis the limma model was then fitted and t-statistics were computed using the Empirical Bayes method with the adjusted *p*-value threshold of 0.05. Mean-difference plots were generated with limma in R. Gene ontology analysis was performed by applying the BH method within the R package clusterProfiler.

### Bisulfite amplicon sequencing

To further explore DNAm changes at genomic regions with prominent culture-associated CpGs we used barcoded bisulfite amplicon sequencing (BA-seq) [11, 14]. gDNA was isolated from 2 × 10^6 ^T cells with the DNeasy Blood & Tissue Kit (Qiagen), quantified with a Nanodrop 2000 Spectrophotometer (Thermo Scientific) and a total of 200–500 ng genomic DNA was used for bisulfite conversion with the EZ DNA Methylation Kit (Zymo Research). Target sequences in the genomic regions of *TOX*, *SMAD3* and *GRAP2* were amplified by PyroMark PCR kit (Qiagen) with primers that contain a handle sequences (Supplemental Table S3). PCR products were combined in an equimolar composition followed by a clean-up step using the Select-a-Size DNA Clean & Concentrator Kit (Zymo Research) and diluted with 15% PhiX spike-in control to obtain a 20 picomolar DNA library, which was then 250 bp paired-end sequenced on a MiSeq system with Miseq reagents (Illumina). The Bismark tool was used to map bisulfite converted sequence reads and to determine DNAm levels for each CpG based [21]. On average, 1000 reads could be aligned per genomic region in a sample. Genomic coordinates for hg19, including reference genes and CpG locations, were obtained from UCSC and plotted with the R package Gviz.

## Results

### Cultivation of T cells is associated with discrete DNA hypermethylation

**Fig. 1:**
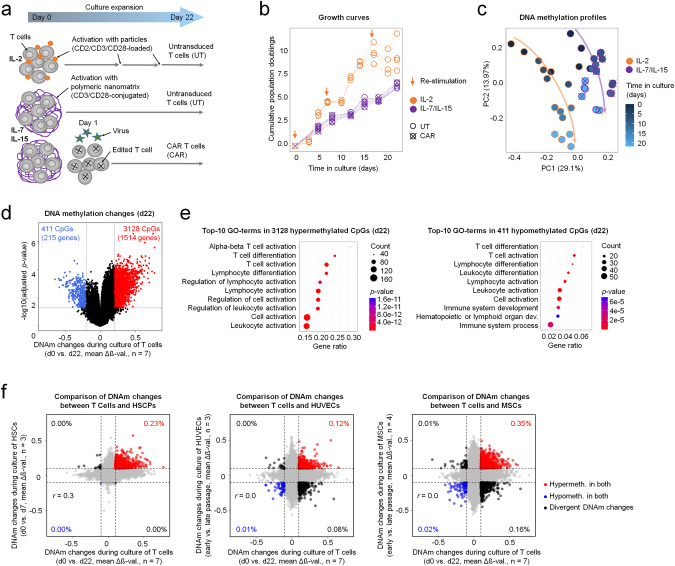
CAR T cells accumulate DNA methylation changes during culture expansion. **a** Different culture conditions for expansion of untransduced T cells (UT) and CAR T cells for up to 22 days. **b** Growth curves of UT and CAR T cells during small scale in vitro expansion. Arrows indicate (re-)stimulation of T cells with particles. **c** Principal component analysis (PCA) of DNA methylation profiles. The arrows indicate trajectories with culture time (shades of blue) for the different culture conditions. **d** Volcano plot showing differentially methylated CpG sites (hypermethylated in red, hypomethylated in blue) in d0 (UT) *versus* d22 samples (UT and CAR). **e** Bubble plots of the top-10 Gene Ontology (GO) terms for significantly hyper- and hypomethylated CpGs after 22 days of culture expansion (Gene ratio = differentially methylated/total number genes in this pathway; bubble size = absolute number of genes in the set; color code depicts significance level). **f** The scatter plots compare mean methylation changes (mean Δß-value) during in vitro expansion in T cells (UT and CAR; d0 versus d22) as compared to culture of hematopoietic stem and progenitor cells (HSPCs), human umbilical vein endothelial cells (HUVECs), or mesenchymal stromal cells (MSCs), respectively. The percentages of CpG sites with more than 10% mean DNAm change are highlighted (red = hypermethylated in both cell types; blue = hypomethylated in both cell types; black = divergently methylated; *r* = Pearson correlation coefficient). Here, all CpGs that were shared between the 450 K and EPIC platform are depicted.

To gain insight into epigenetic modifications during expansion of CAR T cells, independent of specific manufacturing conditions, we considered different cell culture regimen for expansion of T cells with or without transduction with the CAR vector for up to 22 days (Fig. 1a). Expansion with IL-2 and repeated stimulation with CD2/CD3/CD28-loaded particles facilitated higher population doublings, but it was also accompanied by increased cell death, particularly after the second re-stimulation, which is in line with previous findings [22]. In contrast, stimulation of cells with IL-7, IL-15, and a polymeric nanomatrix conjugated with CD3/CD28 enabled almost linear expansion rates with a doubling time of about 3.3 days (Fig. 1b). Flowcytometric analysis showed an immunophenotypic shift towards a central memory phenotype (T_CM_; CD62L^+^ CD95^+^ CD45RO^+^), particularly between day 8 and day 15, and increasing stem cell memory phenotype (T_SCM_; CD62L^+^ CD95^+^ CD45RO^-^), independent of CAR transduction (Fig. S1a, b). Of note, the ratio of CD4^+^ *versus* CD8^+^ T cells declined (Fig. S1c), whereas the fraction of cells with CAR-surface expression remained relatively stable during culture expansion of CAR T cells (Fig. S1d, e). Overall, these data illustrate considerable immunophenotypic changes during culture expansion.

Next, we analyzed DNAm profiles of untransduced T cells (UT) and CAR T cells with Illumina EPIC Bead Chips. Epigenetic deconvolution of leukocyte subsets was in line with the flow cytometric measurements and showed a decline of the CD4^+^ *versus* CD8^+^ T cell ratio during culture (Fig. S2a). For further analysis, we therefore excluded CpGs that were significantly differentially methylated between CD4^+^ and CD8^+^ T cells to avoid a cell-type-related bias (adjusted *p* < 0.05 or DNAm difference >10%; Fig. S2b, c). Unsupervised principal components analysis (PCA) indicated continuous DNAm pattern changes during culture (Fig. 1c). The second component (PC2) showed even linear correlation with the cumulative number of population doublings (cPD; Fig. S2d). A pairwise comparison of UT and CAR T cells did not reveal significant DNAm changes that are attributed to the CAR transduction (adjusted *p* < 0.05; Fig. S2e). However, clear epigenetic differences between different culture conditions could be seen: 1121 CpGs were hypermethylated and 90 CpGs were hypomethylated in T cells expanded with IL-2, when compared to IL-7/IL-15 (mean DNAm difference >20% and adjusted *p* < 0.05; Fig. S2f).

When we compared DNAm changes between day 0 (d0) and day 22 (d22) preparations, there were even more pronounced differences, with 3128 hypermethylated and 411 hypomethylated CpG sites (Fig. 1d; Supplemental Table S4). These CpGs were associated with 1514 and 215 different genes, respectively. Gene set enrichment analysis demonstrated that the cultivation-time-associated hypermethylation was highly enriched in functional categories of leukocyte and T cell activation, whereas hypomethylation was enriched in categories for differentiation of T cells and leukocytes (Fig. 1e). To understand if the epigenetic modifications during expansion of T cells are cell type specific, we compared them with culture-associated DNAm of hematopoietic stem and progenitor cells (HSPCs) [13], human umbilical vein endothelial cells (HUVECs) [14], or mesenchymal stem cells (MSCs) [15]. Despite the different culture time, culture conditions, and analysis platforms, there was a correlation of culture-associated DNAm changes in HSPCs and CAR T cells (Pearson correlation coefficient *r* = 0.3), while this was hardly observed for the other less related cell types (Fig. 1f). Taken together, culture expansion of T and CAR T cells is associated with continuous and pronounced DNAm changes, particularly in genes that are relevant for T cell function.

### T cell expansion interferes with T cell regulatory networks

**Fig. 2:**
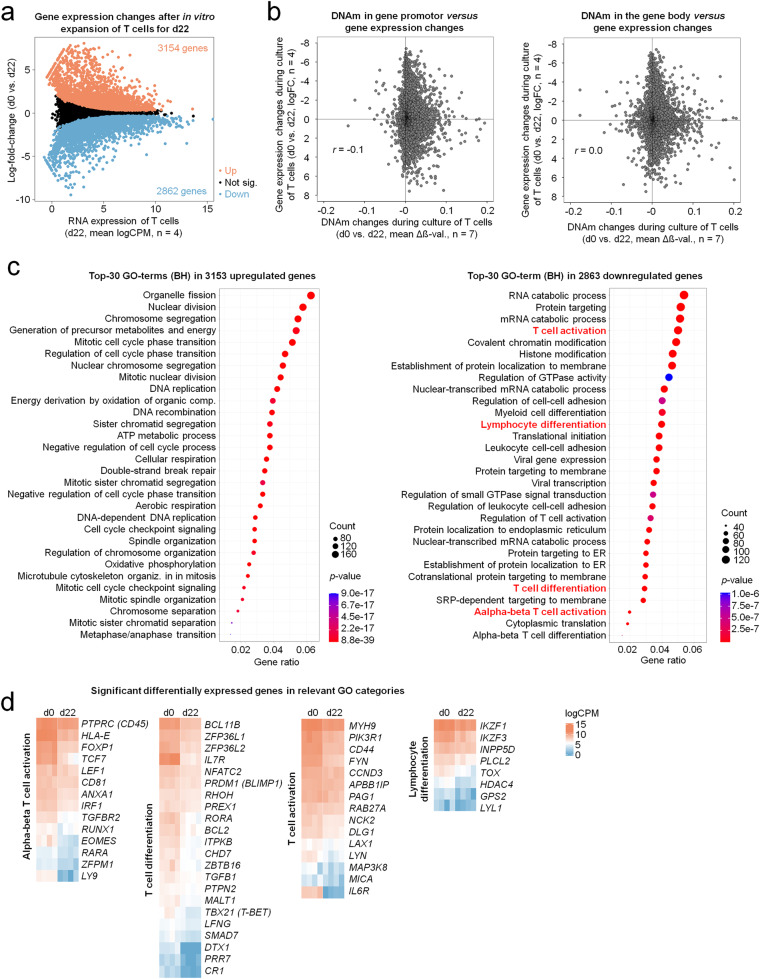
Culture expansion of T cells leads to down-regulation of relevant genes for T cell function. **a** Mean average (MA) plot of RNA-Seq data illustrating differentially expressed genes in T cells after 22 days of culture expansion (adjusted *p* < 0.05, *n* = 4). **b** Correlation of gene expression changes with DNA methylation changes during culture expansion (day 0 versus day 22) for CpGs that are localized on promoter regions (left) or in the gene body (right) of the corresponding genes. If multiple CpGs occurred in these regions the mean DNAm was calculated to provide a single dot per gene. **c** Gene Ontology classification of differential gene expression upon expansion of T cells shown as bubble plots of the top 30 GO-terms ranked by gene ratio for significantly up- and down-regulated genes in T cells after 22 days of culture expansion. For the down-regulated genes four categories are highlighted in red, which revealed also highest enrichment for hypermethylated CpGs (illustrated in Fig. 1e). **d** Heatmap of gene expression levels (indicated as log counts per million; logCPM) of genes that are significantly down-regulated during long-term culture and within these four Gene Ontology categories.

Subsequently, we analyzed if the culture-associated DNAm is also reflected in differential gene expression. RNA sequencing data of d0 *versus* d22 revealed 3154 up- and 2862 down-regulated genes in T cells that were expanded with IL-2 (Fig. 2a; Supplemental Table S5; adjusted *p* < 0.05). Overall, we did not observe a clear association between gene expression and DNAm changes for CpGs that are either in the promoter or gene body (Fig. 2b), which supports the notion that increasing DNAm is not necessarily reflected in down-regulation on gene expression level [23]. Nevertheless, gene ontology analysis (GO) revealed a significant enrichment of down-regulated genes in the categories T cell activation, lymphocyte differentiation, T cell differentiation, and alpha-beta T cell activation (Fig. 2c). Thus, differentially expressed genes were enriched in very similar functional categories as observed for DNAm changes. We further investigated the relevant genes in these overlapping pathways (Fig. 2d). Among the downregulated genes were *TCF1* and *LEF1*, two critical regulators of stemness and memory formation [24]. Likewise, the two chemokine receptors *IL6R* and *IL7R* were downregulated, which are involved in stimulation of naïve precursor T cells [25]. Furthermore, they are crucial mediators of anti-tumor activity as well as cytokine release syndrome (CRS) and immune effector cell-associated neurotoxicity syndrome (ICANS), the most frequently observed immune-mediated adverse events following CAR T cell administration [26]. DNA hypermethylation and downregulation of gene expression was also observed for *TOX*, *T-BET*, and *EOMES*, core transcription factors connected to T cell exhaustion in vivo [27].

### Cultivation-associated epigenetic drift in differentially methylated regions

**Fig. 3:**
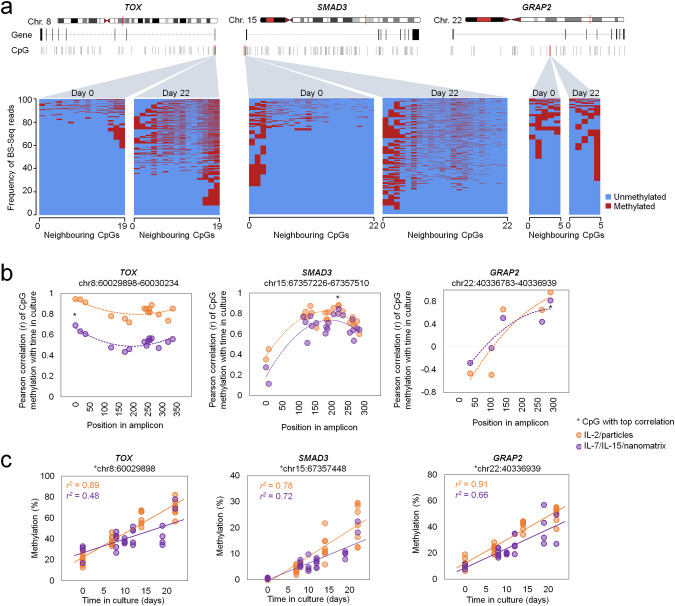
Neighboring CpGs stochastically gain methylation during culture expansion. **a** Culture-associated DNA methylation (DNAm) patterns were analyzed in genomic regions of three genes (TOX, SMAD3, and GRAP2) with bisulfite amplicon sequencing (BS-Seq). The heatmaps represent the sequel of methylated and non-methylated sites within individual reads of the three amplicons, respectively. Results are exemplarily depicted for one sample at day 0 and day 22. **b** Pearson correlation of DNAm at neighbouring CpG sites within the amplicons of TOX, SMAD3, and GRAP2 for T cells expanded with two culture conditions (orange: IL2/particles, *n* = 8; purple: IL-7/IL15/nanomatrix, *n* = 5). The position of CpG sites within the amplicons are depicted (asterisk marks CpG with highest Pearson correlation). **c** The linear correlation of DNAm with culture time is exemplarily depicted for those CpGs with highest correlation.

To gain better insight into DNAm changes at cultivation-time-associated regions, we exemplarily performed targeted bisulfite amplicon sequencing (BS-Seq) for *TOX*, *SMAD3*, *GRAP2*. If the DNAm changes were mediated by a targeted regulatory protein complex with an epigenetic writer, it would probably also coherently modify neighboring CpGs. In contrast, we observed the sequel of methylated or non-methylated CpGs seemed to occur randomly within individual reads of BS-Seq data (Fig. 3a). The stochastic modification therefore indicates that cultivation-associated DNAm in CAR T cells is rather due to epigenetic drift [9, 11]. On the other hand, across different preparations of T cells and CAR T cells the average DNAm levels showed similar gain at neighboring CpGs (Fig. 3b). In fact, the top CpGs showed almost linear increase with time in culture, and this was slightly accelerated in T cells with IL-2 culture condition (Fig. 3c), corresponding to the higher cumulative population doublings as indicated above.

### Epigenetic predictor for time in culture of CAR T cells

**Fig. 4:**
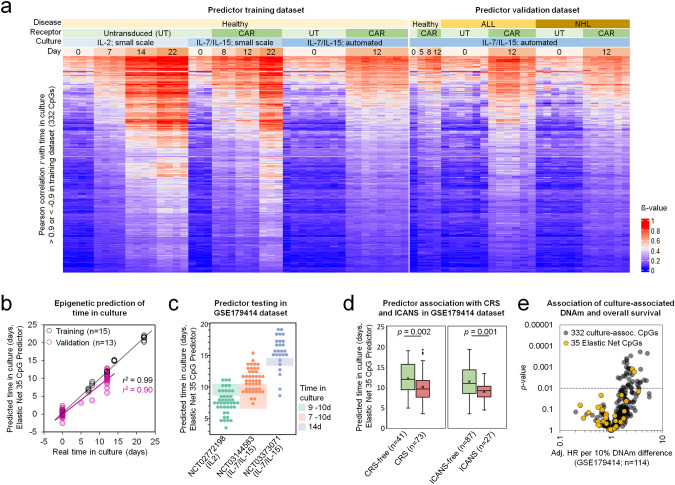
Epigenetic predictions for time in culture expansion of CAR T cells. **a** Heat map showing DNA methylation (DNAm) levels of the 332 CpGs with almost linear gain (Pearson correlation r > 0.9; 336 CpGs) or loss of methylation (*r* < −0.9; 3 CpGs) with time in culture for different T cell preparations in the training set and an independent validation set. DNAm levels (β-values) are indicated by the color code. **b** Epigenetic predictions based on 35 CpGs (elastic-net) correlate with time in culture for the training (r2 = 0.99; *n* = 15) and the validation dataset (r2 = 0.90; *n* = 13). **c** The 35 CpG epigenetic predictor for time in culture was subsequently applied on DNAm profiles of three clinical studies: NCT02772198 (*n* = 43), NCT03144583 (*n* = 45) and NCT03373071 (*n* = 26). The interleukins supplemented during expansion and the real time of culture (shaded areas) are indicated. **d** The DNAm profiles of these three studies were subsequently stratified into those with or without later occurrence of cytokine release syndrome (CRS) or immune effector cell-associated neurotoxicity syndrome (ICANS). The box plot indicates predicted time in culture (interquartile range (IQR) and whiskers denote the 1.5 × IQR; *p* calculated with Student’s *t*-test). **e** The association of DNA methylation levels in individual culture-associated CpGs of CAR T cells was tested with overall survival post transplantation. The scatter plot depicts hazard ratios (HR per 10% DNAm difference) and *p*-values for the 332 culture-associated CpGs (adjusted for disease and clinical trial; not adjusted for multiple testing of 332 CpGs) in the testing dataset of three clinical trials (GSE179414; *n* = 114). Yellow dots demarcate the 35 CpGs of the elastic-net predictor for time in culture.

Since several CpGs showed almost linear DNAm changes with time of culture, we subsequently generated a robust epigenetic signature to align with culture expansion. To this end, we compiled a training dataset consisting of untransduced T cells and CAR T cells, with different culture media, and either manual or automated expansion. We then filtered for CpGs with very high linear correlation between DNAm and time in culture (in total 44 hybridizations; Fig. 4a). This analysis provided 336 hypermethylated and only 3 hypomethylated CpGs (Pearson correlation *r* > 0.9 or *r* <  −0.9, respectively; Supplemental Table S6). It has previously been shown that culture-associated CpGs can overlap with age-associated CpGs [28, 29]. To generate an epigenetic predictor for time in culture of T cells we removed 7 CpGs that revealed correlation with chronological age in blood samples (GSE115278, *r* >0.3 or *r*  < −0.3). This overlap was relatively small and we have therefore further tested our culture-associated DNAm change against another dataset of CD4^+^ T cells from different donor ages [30], which substantiated the notion that culture-associated DNAm changes in T cells are rather independent of donor age (Fig. S3).

Subsequently, we calculated a multivariable elastic-net linear regression model with 10-fold cross-validation and derive a predictor for time in culture based on 35 hypermethylated CpGs (Supplemental Table S1). This predictor revealed a high correlation with days in culture of T cells and CAR T cells in the training dataset (*r*^*2*^ = 0.99) and in the independent validation set of clinical CAR T cells (*r*^*2*^ = 0.90; Fig. 4b). Furthermore, the predictions were overall in line with the culture times from publicly available datasets of CAR T cell products that were expanded for 9–10 days with IL-2 in NCT02772198 (*n* = 43), 7–10 days with IL-7/IL-15 in NCT03144583 (*n* = 45), or 14 days with IL-7/IL-15 in NCT03373071 (*n* = 26; GSE179414; Fig. 4c) [8]. These predictions were hardly affected by chronological age or disease (ALL *versus* NHL; Fig. S4).

We next investigated, if our predictor based on culture-associated DNAm is also indicative for clinical outcome after adoptive T cell transfer – even though the time of culture was rather consistent within each clinical trial. Interestingly, cytokine release syndrome (CRS) and immune effector cell-associated neurotoxicity syndrome (ICANS), which might also reflect higher activity of the transplant, were significantly more frequent in CAR T cell products that were predicted to have shorter time in culture (*p* = 0.002 for CRS; *p* = 0.001 for ICANS; Fig. 4d). A multivariate Cox proportional hazard regression model, adjusting for clinical trial and disease, was used to test the association between predicted time in culture and mortality in (GSE179414). Estimated hazard ratios (HR) with two-sided 95% confidence intervals exhibited no association between predicted time in culture and overall survival (OS; HR = 0.99; CI = 0.8–1.2; *p* > 0.05). Individual analysis of the 35 CpGs, comprising the elastic-net predictor, revealed that two of these CpGs were associated with overall survival (HR > 1; *p* < 0.01, not adjusted for multiple testing; Fig. 4e). Thus, there was an association of epigenetic predictions with ICANS and CRS, whereas only subset of culture-associated CpGs might be related with therapeutic outcome.

### Hypermethylation at specific cultivation associated CpGs is indicative for loss of T cell potential

**Fig. 5:**
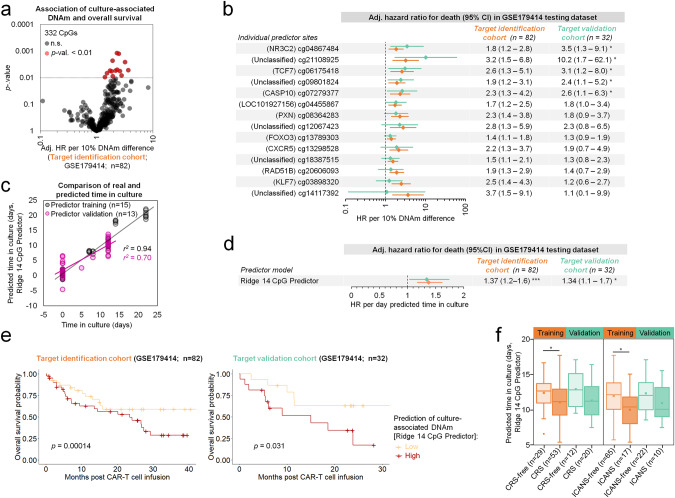
Culture-associated DNA methylation changes in CAR T cells are indicative for therapeutic outcome. **a** The association of cultivation-time-associated DNA methylation (DNAm) changes with overall survival was tested in a target identification cohort comprising three clinical trials (subset of GSE179414; *n* = 82). Multivariate Cox regression analysis revealed that 14 CpG sites out of the 332 CpGs with highest correlation with culture time were associated with overall survival (hazard ratios adjusted for disease and clinical trial (Adj. HR); not adjusted for multiple testing of 332 CpGs). **b** For these 14 CpGs, association with overall survival was further tested for the target identification cohort (orange; *n* = 82) and the target validation cohort (turquoise; *n* = 32). Hazard ratios (adjusted for disease and clinical trial) and confidence intervals (CI) are depicted (^*^p < 0.01). **c** A multivariable epigenetic predictor for culture time based on the 14 CpGs was subsequently trained (*r*^*2*^ = 0.94; *n* = 15) and validated (*r*^*2*^ = 0.70; *n* = 13) in independent datasets (as depicted in Fig. 4a). **d** Cox proportional hazards model (adjusted for disease and clinical trial) for the epigenetic predictions of time in culture based on the 14 CpGs. Longer predicted time in culture of CAR T cells was at higher risk for death (^*^p-value < 0.01; ^***^p < 0.0001). **e**. Kaplan–Meier estimates of overall survival for the target identification (*n* = 82) and validation cohorts (*n* = 32). Subgroups were divided into low and high estimates of culture-associated DNAm based on the 14 CpG predictor. **f** Box plot showing the comparison of predicted time in culture between patient cohorts without/with cytokine release syndrome (CRS) or immune effector cell-associated neurotoxicity syndrome (ICANS). Boxes indicate the interquartile range (IQR) and whiskers denote the 1.5 × IQR (^*^p < 0.01).

We further investigated the association of DNAm at the 332 cultivation-time-associated CpGs with overall survival. As clinical metadata was only available for samples from three clinical trials (GSE179414: NCT03144583, NCT02772198, and NCT03373071), we randomly divided this dataset into a target identification cohort of 82 patients and a target validation cohort of 32 patients (Supplemental Table S7) with equal distribution regarding age, clinical trial, disease, clinical response, and CRS/ICANS. Multivariate Cox regression analysis in the target identification cohort revealed that 14 CpG sites out of the 332 cultivation-time-associated CpGs were associated with a higher patient death rate (HR > 1, *p* < 0.01; Fig. 5a). It has to be noted that after correction for multiple testing (for 332 CpGs) none of the individual CpGs would have reached statistical significance (Supplemental Table S2). However, five of the 14 CpGs were also associated with overall survival in the target validation cohort, despite the relatively small sample size (Fig. 5b) and three of these CpGs were associated with the genes *NR3C2*, *TCF7* and *CASP10*. The effect of DNAm on overall survival was also evident in Kaplan-Meier survival plots where the samples were classified into quartiles according to DNAm levels (Fig. S5).

To derive an epigenetic predictor that is indicative for loss of therapeutic potential, we subsequently trained a model based on the 14 culture-time-associated CpGs that might be associated with overall survival. This model was trained on our initial training dataset of T cells and CAR T cells during culture expansion (multivariable ridge linear regression model with 10-fold cross-validation; Supplemental Table S2). This model showed again high correlation between predicted and real time in culture in the training set (*r*^*2*^ = 0.94) and in the independent predictor validation set (*r*^*2*^ = 0.70; Fig. 5c). We subsequently used this ridge 14 CpG model to test for association with overall survival in the clinical dataset of the target identification (*n* = 82; HR = 1.37; *p* < 0.0001) and in the target validation cohort (*n* = 32; HR = 1.34; *p* < 0.01; Fig. 5d). The clear association of this epigenetic signature for culture expansion with OS was also evident in the Kaplan-Meier plots (Fig. 5e).

The time of CAR T cell expansion was relatively consistent within each of the three clinical trials (NCT03144583, NCT02772198, and NCT03373071) and thus the epigenetic predictions do not only reflect culture time, but also sample-intrinsic differences. As anticipated the predicted time in culture varied between studies according to different production regimen (Fig. S6a), while overall survival did not significantly differ between the clinical trials (Fig. S6b). Notably, the epigenetic predictions were also associated with overall survival within individual clinical trials, despite the relatively small sample sizes (Fig. S6c). Furthermore, the CAR T cell products of patients that suffered from CRS or ICANS upon treatment were overall predicted to have less culture expansion (*p* < 0.01 in target identification cohort; Fig. 5f). Thus, cultivation-time-associated DNAm changes in the CAR T cell transplants are related to clinical outcome post infusion.

## Discussion

Manufacturing of CAR T cells is a complex multi-step process, which should be further optimized to increase therapeutic outcome. Our study demonstrates that in vitro expansion of T cells evokes DNAm changes at specific sites in the genome, and this seems to be accompanied by reduced long-term survival upon CAR T cell product administration. Another recent study described the EPICART epigenetic signature, which is also indicative of clinical outcome [8]. Notably, there was no overlap between the 18 CpGs of EPICART with either the 35 CpGs of our epigenetic predictor of time in culture, or the 14 CpGs of the loss of T cell potential predictor. This may be expected, since EPICART was trained on DNAm changes associated with the transduction of the CAR vector–and hence not related to our cultivation-time-associated DNAm changes. The results of this study further demonstrate that epigenetic biomarkers are powerful tools to further optimize the CAR T cell manufacturing process and to gain insight into the molecular mechanisms that are relevant for the therapeutic success.

We observed that most of the culture-associated DNAm changes were hypermethylations–particularly at those CpGs with continuous changes throughout culture expansion. Notably, very similar DNAm changes were observed during expansion of T cells and CAR T cells, with different culture media, and with manual small scale or automated large-scale production. If this hypermethylation was relevant for the reduced therapeutic outcome, it might be advantageous to block de novo DNAm during CAR T cell expansion. In fact, it was recently shown that low-dose priming with decitabine - an inhibitor of DNA methyltransferases (DNMTs) - decreased exhaustion, maintained memory phenotype and effector functions, and enhanced antitumor response in mice [31, 32]. Furthermore, in mice it has been demonstrated that de novo DNAm by Dnmt3a controls early effector CD8^+^ T-cell fate decisions following activation [33, 34]. We have recently demonstrated that knockout of DNMT3A prevents almost the entire de novo DNAm during hematopoietic differentiation of into induced pluripotent stem cells (iPSCs) [35]. Another study has recently shown that deletion of DNMT3A in human CAR T cells prevents exhaustion and enhances anti-tumor activity [36]. Notably, these studies have also highlighted marked changes in similar genes as observed in our study, including *TCF7* and *LEF1*. Thus, we anticipate that the knockout of DNMT3A is in fact blocking the culture-associated DNAm changes during CAR T cell expansion, which resulted in improved CAR T cell functionality.

The same epigenetic process might even contribute to the exhaustion of CAR T cells post infusion. The relevance of epigenetic regulation for CAR T cell function in vivo is exemplified by a patient report with clonal CAR T cell expansion with biallelic TET2 disruption [37]. Efforts have been channeled to maintain CAR T cells in naïve or memory stem cell fate, to improve anti-tumor effects and long term persistence in vivo [38, 39]. Notably, a recent study investigated longitudinal DNAm changes in CAR T cells post infusion and identified continuously increasing epigenetic changes that may be directly associated with exhaustion, including repression of *TCF7* and *LEF1* and demethylation of *TOX* [40]. This striking overlap supports the hypothesis that the DNAm changes, which are continuously acquired during culture expansion, continue also after transfusion and contribute to CAR T cell exhaustion.

Unfortunately, the term “T cell exhaustion” combines a heterogeneous group of mechanisms, which remain poorly defined [41]. CAR T cell dysfunction is mainly characterized by immunophenotypic changes; however, molecular phenotyping might be more appropriate. It was suggested that dynamic changes in chromatin accessibility (analyzed by ATAC-seq) and three-dimensional chromosome conformation precede changes in gene expression, particularly proximal to exhaustion-associated genes [42]. A notable example is the *TOX* gene that was found to be a driver of T cell exhaustion in mice [43]. Furthermore, several genes with hypermethylation during culture-expansion are relevant to maintain a naïve or memory-like phenotype of T cells, including i.e. *TCF7*, *FOXO3*, and *PXN* [44, 45]. Thus, the epigenetic modifications during culture expansion in vitro may reflect CAR T cell exhaustion – or they might even be related to replicative senescence.

Primary cells can only be culture expanded for a limited number of passages before they stop proliferation and enter a state of replicative senescence. For other cellular therapeutics, such as MSCs and HSPCs, it is well known that in vitro expansion results in continuous decline in proliferation, differentiation potential, and therapeutic potential [46, 47]. Interestingly, the long-term culture-associated DNAm changes in MSCs and fibroblasts can be completely reversed by reprogramming iPSCs, and they are then gradually reacquired upon re-differentiation [15, 48]. Here, we demonstrate that the culture-associated DNAm changes in CAR T cells are rather related to those occurring in HSPCs, which supports the notion that there are differences in the molecular changes of hematopoietic and mesenchymal lineages. Such signatures may also be affected by changes in the cellular composition, and we have therefore excluded CpGs that might rather reflect the changes in the CD4^+^/CD8^+^ ratio or chronological age. However, it remains largely unclear how culture-associated DNAm changes are regulated.

When we analyzed culture-associated DNAm changes in CAR T cells by targeted amplicon sequencing, we observed that the neighboring CpGs are not coherently modified on the same DNA strand. This may be unexpected, since a directed epigenetic regulation by binding of an epigenetic writer would most likely also modulate the neighboring CpGs of the differentially methylated genomic region [49]. Thus, the culture-associated DNAm changes in CAR T cells may rather reflect dysregulation, as suggested for epigenetic drift during aging of the organism [50, 51]. A better understanding of how DNAm changes evolve in culture expansion might shed light into the underlying process and provide more elegant therapeutic mechanisms to generate improved therapeutic CAR T cell products and to prevent their exhaustion.

We demonstrated that the DNAm level at various culture-associated CpGs in CAR T cells is significantly associated with overall survival post infusion. Our 14 CpG signature may provide a powerful predictor, not only to estimate therapeutic response, but also to further fine tune culture conditions for a better therapeutic outcome. It has to be noted that within each of the clinical trials the culture time was rather consistent – hence, our loss of T cell function predictor captures individual variation between CAR T cell products rather than only time in culture. These changes may either derive from patient specific variation, different pretreatment, or from differences during the manufacturing process of the CAR T cell product. It is a limitation of our current study that important covariates for disease status, chemotherapeutic regimen, and CAR T cell production procedures were not available and these important aspects need to be addressed in the future. This will be important to better understand what contributes to the epigenetic differences in addition to culture-time. Furthermore, it should be explored if the signature might even be indicative on the starting material before CAR T cell production–which would be an enormous benefit to save time and costs during treatment.

Our results clearly suggest that a shortened cell culture time during manufacturing might be advantageous, but whether very short in vitro culture of CAR T cells really results in better clinical outcome also remains to be proven by appropriate clinical trials. In fact, there may be a paradigm shift to reduce the culture expansion of CAR T cells. Since the FDA-approval of the first CAR T cell therapy in 2017 there is a growing perception that the phenotype is more important than absolute cell numbers. While infusion of higher numbers of effector T cells might induce higher initial response rates, long term in vivo persistence of stem-like CAR T cells is required to avoid relapse [52]. When mice were treated with CAR T cells generated without in vitro activation and expansion, their response was better than with CAR T cells that were produced with standard protocols [53]. Recently, multicenter phase I studies have been initiated to evaluate the safety and preliminary efficacy of CAR T cells that have been manufactured in less than two days, in patients with diffuse large B-cell lymphoma (NCT03960840) or multiple myeloma (NCT04318327). Thus, it will be interesting to analyze if shortened manufacturing will improve the therapeutic efficiency.

Several recent studies indicated that inhibition of de novo methylation during CAR T cell expansion may block aberrant DNAm and thereby increase therapeutic efficacy. The results of our current study provide another puzzle piece, substantiating that these detrimental DNAm changes are continuously acquired during culture expansion of the manufacturing process. Our data support the notion that manufacturing process design is an important contributing aspect of CAR T cell treatment failure. A shortened cultivation period to avoid dysfunctional methylation programs might be a promising manufacturing strategy to improve therapeutic efficacy of adoptive cell therapies. Our epigenetic signature for loss of T cell potential provides a biomarker for quality control of CAR T cell productions and to identify patients at risk for adverse events.

## Supplementary information

Supplemental Information

Supplemental Table 4

Supplemental Table 5

Supplemental Table 6

## Data Availability

The EPIC BeadChip data and RNA-seq data of in vitro expanded T cells are available in the GEO repository with the accession number GSE220581. Furthermore, we utilized previously published Illumina 450 K and EPIC BeadChip datasets that are accessible at GEO under the accession numbers GSE179414 (CAR T cells) [8], GSE110554 (flow-sorted T cells) [54], GSE67705 (CD4^+^ T cells) [30], GSE82234 (HUVECs) [14], GSE37066 (MSCs) [15], GSE115278 (PBMCs) [55], and GSE40799 (CD34^+^ HSPCs) [13].
