# Supplementary material for: Culture expansion of CAR T cells results in aberrant DNA methylation that is associated with adverse clinical outcome
Source: Leukemia. 2023 Jul 14;37(9):1868–78. doi: 10.1038/s41375-023-01966-1 (PMC10457202; doi:10.1038/s41375-023-01966-1)
Supplement: Supplementary file 1 — Supplemental Information [file 41375_2023_1966_MOESM1_ESM.pdf]

Supplementary Materials

Culture expansion of CAR T cells results in aberrant DNA methylation that is associated with adverse clinical outcome

Lucia Salz, Alexander Seitz, Daniel Schäfer, Julia Franzen, Tatjana Holzer, Carlos A. Garcia-Prieto, Iris Bürger, Olaf Hardt, Manel Esteller, Wolfgang Wagner

Table of contents

Supplementary Figures 2
- Figure S1. Immunophenotypic analysis during small scale expansion for up to 22 days. 2
- Figure S2. Filtering strategy and analysis of DNA methylation changes. 3
- Figure S3. Comparison of age- and culture-associated DNAm changes in T cells. 4
- Figure S4. Association of the 35CpG predictor with clinical data. 5
- Figure S5. Kaplan-Meier estimates of overall survival for DNAm at five culture-associated CpG sites. 6
- Figure S6. Association of epigenetic predictions with overall survival within individual clinical trials. 7

Supplementary Tables 8
- Table S1. Predictor for time in culture (35 CpGs, elastic net). 8
- Table S2. Survival-associated predictor for time in culture (14 CpGs). 9
- Table S3. Primers for bisulfite amplicon sequencing. 9
- Table S4. Differentially methylated CpGs during culture expansion. 10
- Table S5. Differentially gene expression during culture expansion. 10
- Table S6. CpGs with linear DNAm changes during culture expansion. 10
- Table S7. Distribution of clinical samples for training and validation sets. 11

Supplementary References 12

Supplementary Figures

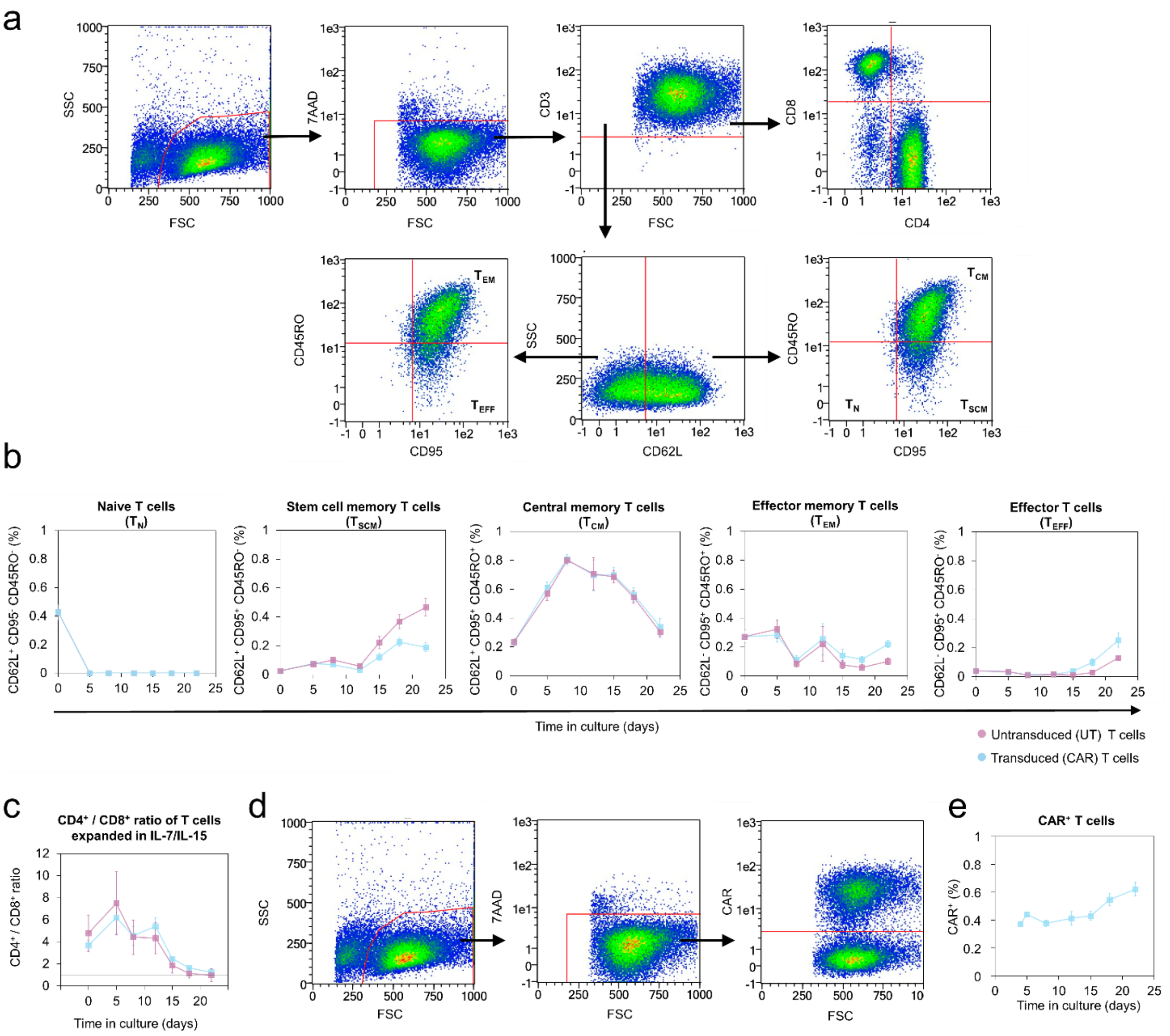

Figure S1. Immunophenotypic analysis during small scale expansion for up to 22 days.

a. Representative gating strategy for T cells (CD3$^+$); CD4$^+$ and CD8$^+$ subpopulations; Naïve: CD62L$^+$,CD95$^-$, and CD45RO$^-$; Stem cell memory (T_{SCM}): CD62L$^+$, CD95$^+$, and CD45RO$^-$; Central memory (T_{CM}): CD62L$^+$, CD95$^+$ and CD45RO$^+$; Effector memory (T_{EF}): CD62L$^-$, CD95$^+$, and CD45RO$^+$; Effector (T_{EFF}): CD62L$^-$, CD95$^+$, and CD45RO$^-$.

b. Changes in the proportion of different T cell subsets during culture expansion (with IL-7/IL-15/nanomatrix; blue = Untransduced (UT) T cells; purple = CAR T cells; n = 3).

c. Exemplary changes in the ratio of CD4$^+$ *versus* CD8$^+$ T cells expanded in same culture conditions with IL-7/IL-15-supplemented medium (n = 3). A similar shift in favor of CD8+ T cells was observed with IL-2-supplemented culture medium (data not shown).

d. Representative gating strategy for analysis of CAR surface expression in transduced T cells.

e. Summary plot of transduced T cells expressing CAR on their surface at different time points during culture expansion (means ± SEM).

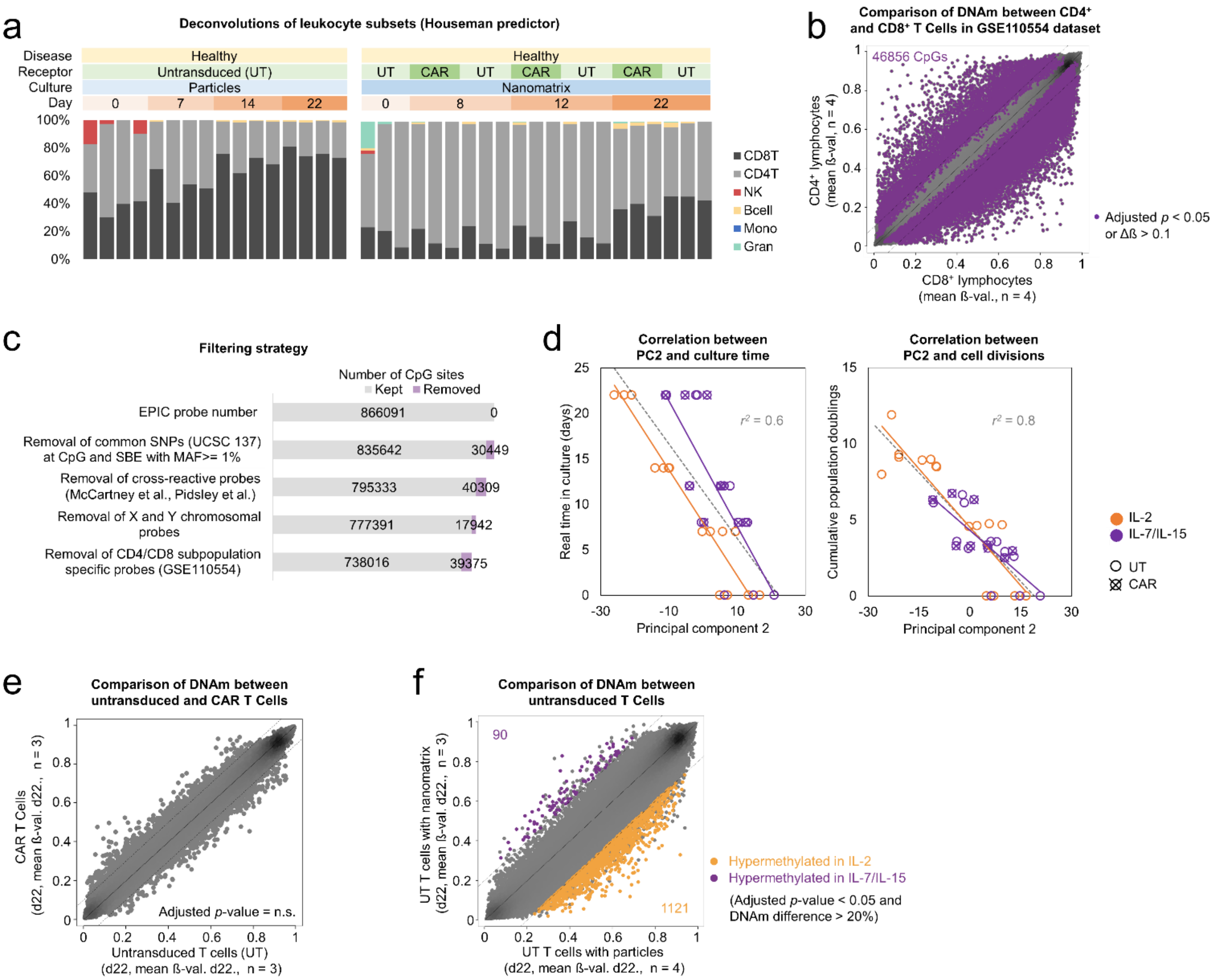

Figure S2. Filtering strategy and analysis of DNA methylation changes.

a. Predicted percentage of blood cell types as estimated by the Houseman algorithm(1) in EPIC BeadChip datasets of T cells from healthy donors; with or without CAR-transduction, culture expanded with the two different culture regimen, and for different time (up to 22 days). The DNA methylation (DNAm) profiles were correctly assigned as T cells with decreasing $CD4^+/CD8^+$ ratio, in analogy to the immunophenotypic analysis.

b. To reduce effects of cellular composition, we exclude CpGs with significant DNAm differences between $CD4^+$ and $CD8^+$ T cells. To identify these CpGs, we utilized a public datasets (GSE110554)(2) to select CpGs with a mean DNAm difference > 10% or an adjusted limma $p < 0.05$; 46,856 CpGs; purple dots).

c. Filtering strategy of CpGs for further analysis of culture-associated DNAm changes. Of the CpGs represented by the EPIC BeadChip we excluded CpGs with SNPs, cross-reactive probes(3, 4), autosomes, and the 46,856 CpGs with differential DNAm between $CD4^+$ and $CD8^+$ T cells.

d. The principal component 2 in the PCA of DNAm profiles in Fig. 1c correlate with time in culture.

e. Pairwise comparison of mean DNAm differences between untransduced (UT) and CAR T cells on day 22 (n = 3; all expanded with IL-7/IL-15/nanomatrix). There were no significant differences (limma adjusted $p <$ 0.05).

f. Scatter plot of DNAm differences between untransduced T cells that were either cultured with IL-2/particles or IL-7/IL-15/nanomatrix for 22 days. The number of significant CpGs are indicated win purple and yellow, respectively (mean difference in DNAm level > 20% and limma adjusted $p < 0.05$).

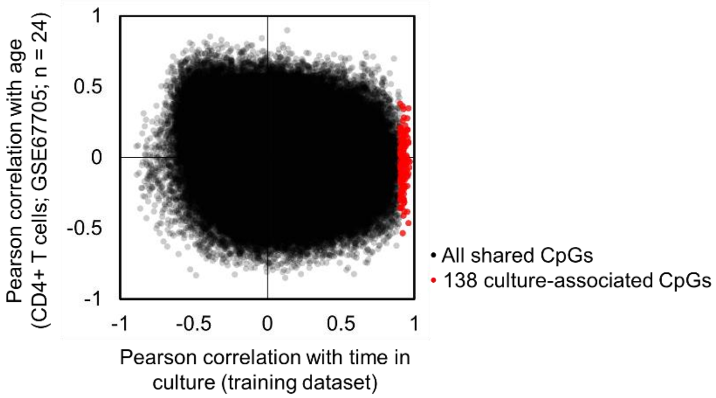

Figure S3. Comparison of age- and culture-associated DNAm changes in T cells.

To further investigate if culture-associated DNAm changes are related to age-associated DNAm changes in T cells, we used DNAm profiles of flow-sorted $CD4^+$ cells from healthy controls (GSE67705; n = 24; age range: 26 – 66 years). This dataset was analyzed with the 450K BeadChip and therefore we focused on the 386,700 CpGs that were shared with the EPIC platform. Of our 339 culture associated CpGs, 138 CpGs in total were also presented by the 450K BeadChip and these are highlighted in red.

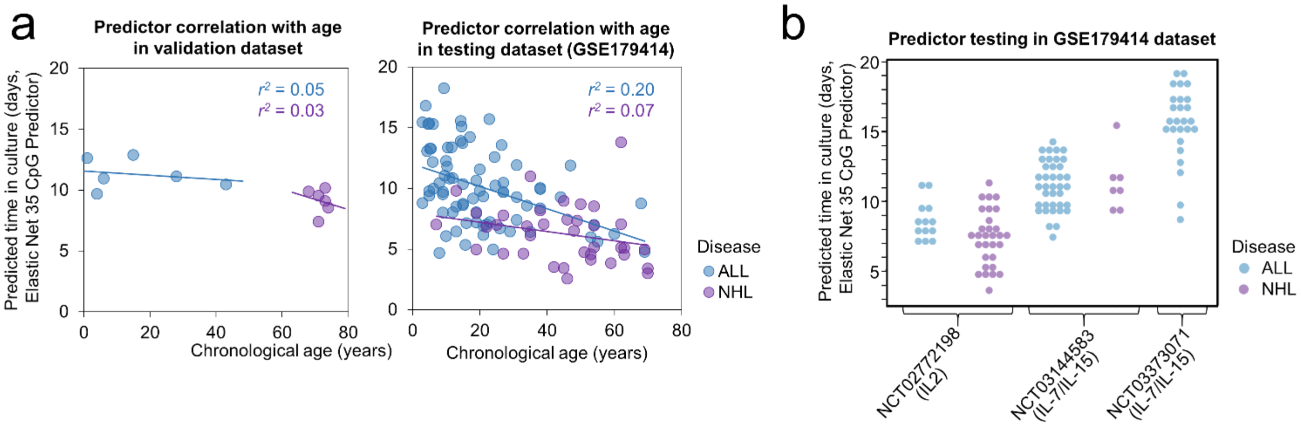

Figure S4. Association of the 35CpG predictor with clinical data.

a. Scatter plot demonstrating that epigenetic predictions of time in culture with the 35 CpG Elastic Net predictor did hardly correlate with chronological age in validation dataset (from Fig. 4a; n = 12) and the additional testing dataset (GSE179414 from Fig. 4c; n = 114). Blue dots = CAR T cells for acute lymphocytic leukemia (ALL) patients; purple dots = CAR T cells for non-Hodgkin lymphoma (NHL) patients.

b. Epigenetic predictions of time in culture based on the 35 CpG elastic net model were stratified by disease (ALL *versus* NHL).

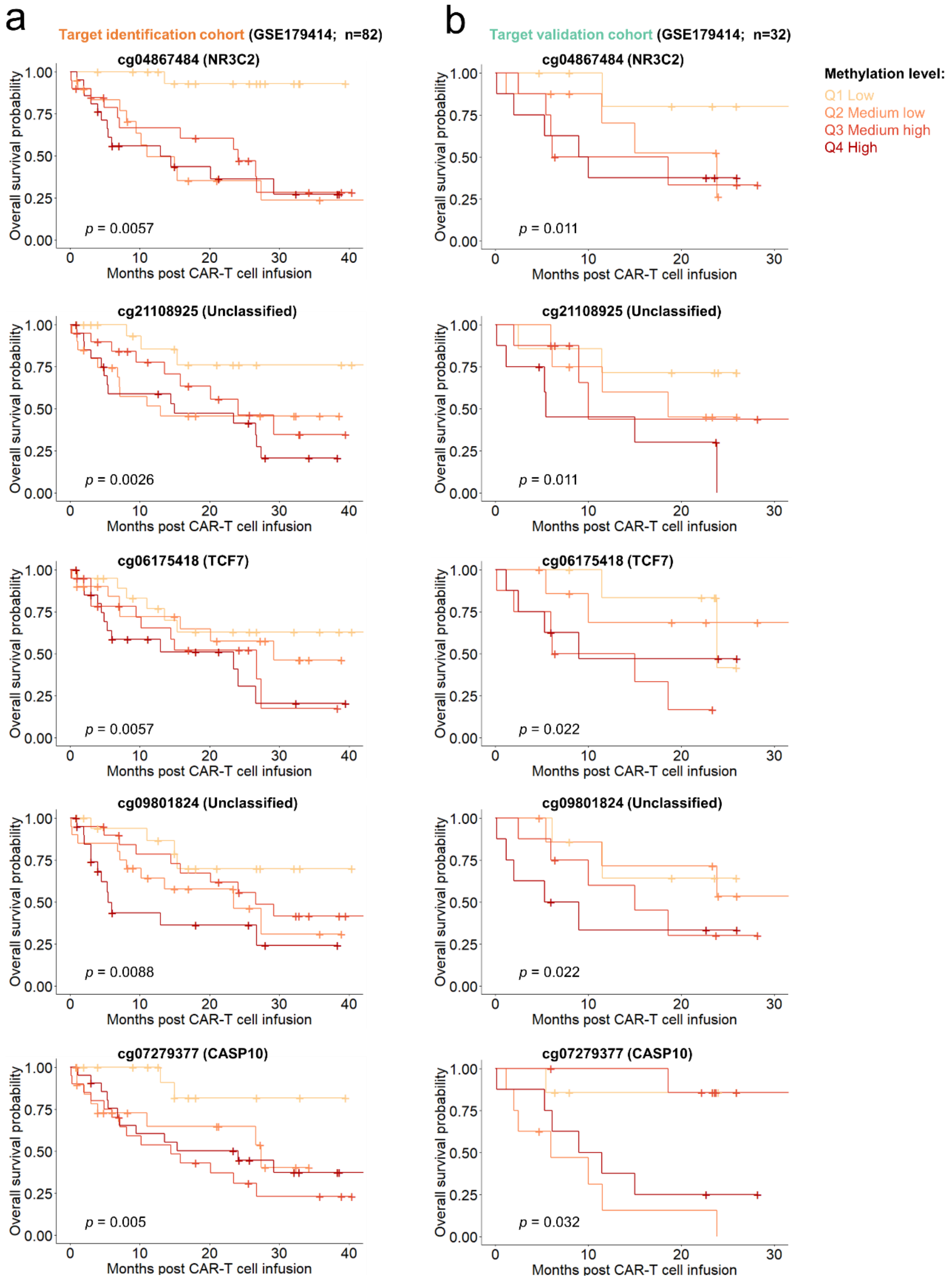

Figure S5. Kaplan-Meier estimates of overall survival for DNAm at five culture-associated CpG sites. The multivariate Cox regression analysis of the target identification (**a**; n = 82) and validation cohorts (**b**, n = 32) indicated that five cultivation time associated CpGs were associated with a higher death rate in the independent target validation cohort. Here, the Kaplan-Meier estimates are provided for these subcohorts, where the patients were divided into quartiles based on the DNAm levels. Wald test *p*-values are illustrated.

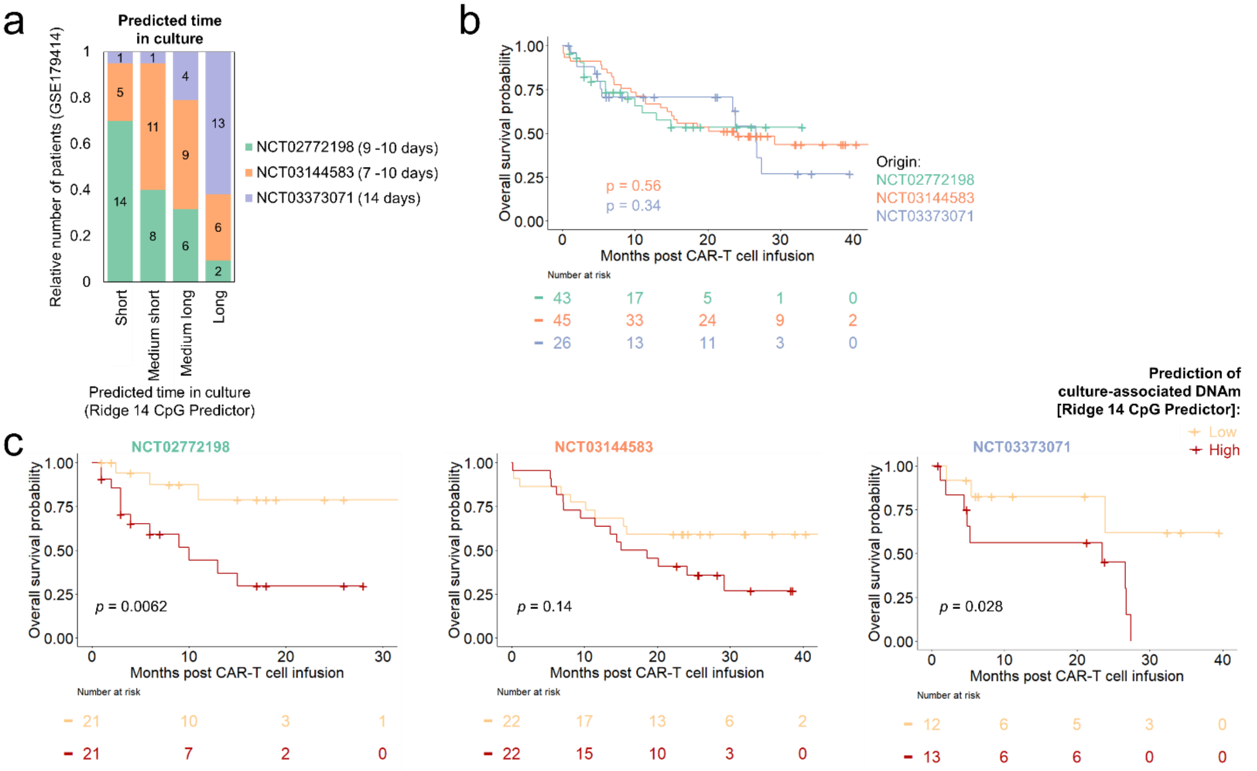

Figure S6. Association of epigenetic predictions with overall survival within individual clinical trials.

a. Stacked bar graph of patient distribution per clinical trial according to predicted time in culture, categorized into quartiles as short, medium short, medium long, and long predicted time in culture. The longer time in culture of NCT03373071 (14 days expansion) is also reflected by higher estimates in the upper quartile (precise information on the exact culture time for each sample are not available for NCT02772198 and NCT03144583).

b. Kaplan-Meier estimates of overall survival comparing the three clinical trials. There were no significant differences in outcome between these trials (here exemplarily depicted as compared to NCT02772198).

c. Kaplan-Meier estimates of overall survival for short *versus* long predicted time in culture for each individual clinical trial (log-rank *p*-values are provided).

Supplementary Tables

Table S1. Predictor for time in culture (35 CpGs, elastic net).

Target site	Gene Name	Gene Group	Coefficient	Methyl. change
(Intercept)			-8.49222	
cg14417920	Unclassified		-4.86	hypo
cg07238065	MED15	Body	0	hyper
cg10599446	Unclassified		0.12	hyper
cg27506442	LOC100188949	TSS1500	0.12	hyper
cg05026393	MTSS1	Body	0.3	hyper
cg06175418	TCF7	Body	0.31	hyper
cg05286545	Unclassified		0.48	hyper
cg13583535	MBP	Body	0.52	hyper
cg20660171	ZFP36L2	TSS1500	0.54	hyper
cg18042851	ISOC1	Body	1.08	hyper
cg11449005	LOC101929705	Body	1.3	hyper
cg09138315	SPOCK2	Body	1.37	hyper
cg09400236	FAM53B	Body	1.41	hyper
cg17544208	RGS3	Body	1.41	hyper
cg11739633	MEF2D	5'UTR	1.42	hyper
cg04715245	IL6R	TSS1500	1.59	hyper
cg19450830	LINC00548	TSS1500	1.68	hyper
cg10939783	SVIL	5'UTR	1.7	hyper
cg03223582	CABIN1	Body	2.2	hyper
cg11641450	RPTOR	Body	2.29	hyper
cg19459094	GRK6	Body	2.46	hyper
cg17459743	RSF1	Body	2.51	hyper
cg22108567	MIR589	TSS200	2.58	hyper
cg04944125	NCK2	5'UTR	2.79	hyper
cg17036007	MEF2D	5'UTR	2.88	hyper
cg06825849	LOC342346	Body	3.44	hyper
cg15300730	ZFP36L2	TSS1500	3.52	hyper
cg06681810	SYNGAP1	Body	4.55	hyper
cg06616765	Unclassified		4.6	hyper
cg13002740	LIME1	5'UTR	5.19	hyper
cg22616379	LIME1	5'UTR	5.34	hyper
cg09884613	CD248	TSS1500	6.83	hyper
cg26123925	Unclassified		9.13	hyper
cg02836771	TSC22D4	TSS1500	9.72	hyper
cg01348086	RGS14	Body	13.99	hyper

Table S2. Survival-associated predictor for time in culture (14 CpGs).

Results were calculated from target identification cohort (subset of GSE179414, n=82). HR = hazard ratio per 10% DNAm difference; CI = confidence interval; OS = overall survival; FDR = FDR-adjusted *p*-value derived from Wald test.

Target site	Gene Name	Gene Group	Coefficient	Methyl. change	HR for death (95%CI)	OS *p*-value	OS FDR *p*-value
(Intercept)			-17.47				
cg08364283	PXN	Body	-1.71	hyper	2.3 (1.4 – 3.8)	0.00119	0.19
cg03898320	KLF7	TSS1500	2.97	hyper	2.5 (1.4 – 4.3)	0.00126	0.19
cg20606093	RAD51B	Body	2.98	hyper	1.9 (1.3 – 2.9)	0.00228	0.19
cg21108925	Unclassified		16.56	hyper	3.2 (1.5 – 6.8)	0.00256	0.19
cg07279377	CASP10	Body	6.23	hyper	2.3 (1.3 – 4.2)	0.005	0.19
cg14117392	Unclassified		3.75	hyper	3.7 (1.5 – 9.1)	0.00516	0.19
cg04455867	LOC101927156		5.50	hyper	1.7 (1.2 – 2.5)	0.00536	0.19
cg13298528	CXCR5	Body	6.42	hyper	2.2 (1.3 – 3.7)	0.00544	0.19
cg06175418	TCF7	Body	3.13	hyper	2.6 (1.3 – 5.1)	0.00567	0.19
cg04867484	NR3C2	Body	-1.69	hyper	1.8 (1.2 – 2.8)	0.0057	0.19
cg18387515	Unclassified		1.73	hyper	1.5 (1.1 – 2.1)	0.00673	0.20
cg12067423	Unclassified		7.77	hyper	2.8 (1.3 – 5.9)	0.00831	0.22
cg09801824	Unclassified		-5.32	hyper	1.9 (1.2 – 3.1)	0.00872	0.22
cg13789303	FOXO3	Body	2.11	hyper	1.4 (1.1 – 1.8)	0.00946	0.22

Table S3. Primers for bisulfite amplicon sequencing.

Primer	Chr	Pos. start	Pos. end	Size	Sequence
TOX	8	60029898	60030234	391	
Forward					5`-GGGATTTTTAATATTTGTTTGGTGG-3‘
Reverse					5`-ATAAAACTTCATCACACAAACTCC-3‘
SMAD3	15	67357226	67357510	341	
Forward					5`-GAATTTAATAGATGTTTTTGAGG-3‘
Reverse					5`-AATACACCCTAAAAAAAACCC-3‘
GRAP2	22	40336783	40336939	235	
Forward					5`-TAAGTATTAGATAGTGTGTAGGAG-3‘
Reverse					5`-CCAATAAAAAACAATAATACACCCC-3‘

Table S4. Differentially methylated CpGs during culture expansion.

The Excel table is provided as separated file. It shows the 441 hypomethylated and 3128 hypermethylated CpGs during 22 days of culture expansion (CpG ID, mean DNA methylation day 0, mean DNA methylation day 22, delta DNA methylation day 0 versus day 22, adjusted *p*-value, chromosome, position, UCSC reference gene).

Table S5. Differentially gene expression during culture expansion.

The Excel table is provided as separated file. It shows the 3154 down- and 2862 up-regulated genes during 22 days of culture expansion (Transcript ID, gene symbol, mean log fold change day 0 versus day 22, limma voom adjusted *p*-value).

Table S6. CpGs with linear DNAm changes during culture expansion.

The Excel table is provided as separated file. It shows the 3 hypomethylated and 336 hypermethylated CpGs (CpG ID, Pearson correlation R with time in culture, UCSC reference gene, UCSC reference gene group).

Table S7. Distribution of clinical samples for training and validation sets.

Patient characteristics	Entire cohort GSE179414 (n = 114)	Test cohort for target identification (n = 82)	Test cohort for target validation (n = 32)
Sex, No. (%)			
Male	68 (59.65)	46 (56.10)	22 (68.75)
Female	46 (40.35)	36 (43.90)	10 (31.25)
Median age (range), y	23.5 (3 – 70)	22.4 (3 – 70)	25.8 (4 – 69)
Age, No. (%), y			
<18	42 (36.84)	31 (37.80)	11 (34.38)
18-29	27 (23.68)	19 (23.17)	8 (25.00)
30-59	34 (29.82)	25 (30.49)	10 (31.25)
≥60	11 (9.65)	7 (8.54)	3 (9.38)
Diagnosis, No. (%)			
B-ALL	77 (67.54)	55 (67.07)	22 (68.75)
B-NHL	37 (32.46)	27 (32.93)	10 (31.25)
DLBCL	20 (17.54)	14 (17.07)	6 (18.75)
PMBCL	11 (9.65)	9 (10.98)	2 (6.25)
Follicular lymphoma	4 (3.51)	3 (3.66)	1 (3.13)
Burkitt lymphoma	1 (0.88)	1 (1.22)	0 (0.00)
Mantle cell lymphoma	1 (0.88)	0 (0.00)	1 (3.13)
Response, No. (%)			
Complete response	74 (64.91)	53 (64.63)	21 (65.63)
Partial response	16 (14.04)	11 (13.41)	5 (15.63)
Stable disease	9 (7.89)	6 (7.32)	3 (9.38)
Disease progression	15 (13.16)	12 (14.63)	3 (9.38)
CRS, No. (%)			
Grade 0	41 (35.96)	29 (35.37)	12 (37.50)
Grade 1	46 (40.35)	34 (41.46)	12 (37.50)
Grade 2	13 (11.40)	9 (10.98)	4 (12.50)
Grade 3	8 (7.02)	6 (7.32)	2 (6.25)
Grade 4	4 (3.51)	3 (3.66)	1 (3.13)
Grade 5	2 (1.75)	1 (1.22)	1 (3.13)
ICANS, No. (%)			
Grade 0	87 (76.32)	65 (79.27)	22 (68.75)
Grade 1	11 (9.65)	7 (8.54)	4 (12.50)
Grade 2	5 (4.39)	3 (3.66)	2 (6.25)
Grade 3	6 (5.26)	2 (2.44)	4 (12.50)
Grade 4	5 (4.39)	5 (6.10)	0 (0.00)
Grade 5	0 (0.00)	0 (0.00)	0 (0.00)
Origin of the CAR T cells			
NCT02772198	43 (37.72)	31 (37.80)	12 (37.50)
NCT03144583	45 (39.47)	32 (39.02)	13 (40.63)
NCT03373071	26 (22.81)	19 (23.17)	7 (21.88)

Supplementary References

1. Houseman EA, Molitor J, Marsit CJ. Reference-free cell mixture adjustments in analysis of DNA methylation data. Bioinformatics. 2014;30(10):1431-9.
2. Salas LA, Koestler DC, Butler RA, Hansen HM, Wiencke JK, Kelsey KT, et al. An optimized library for reference-based deconvolution of whole-blood biospecimens assayed using the Illumina HumanMethylationEPIC BeadArray. Genome Biol. 2018;19(1):64.
3. McCartney DL, Walker RM, Morris SW, McIntosh AM, Porteous DJ, Evans KL. Identification of polymorphic and off-target probe binding sites on the Illumina Infinium MethylationEPIC BeadChip. Genom Data. 2016;9:22-4.
4. Pidsley R, Zotenko E, Peters TJ, Lawrence MG, Risbridger GP, Molloy P, et al. Critical evaluation of the Illumina MethylationEPIC BeadChip microarray for whole-genome DNA methylation profiling. Genome Biol. 2016;17(1):208.
